# A non-canonical *Aux/IAA* gene *MsIAA32* regulates peltate glandular trichome development in spearmint

**DOI:** 10.3389/fpls.2024.1284125

**Published:** 2024-02-05

**Authors:** Vaishnavi Amarr Reddy, Jolly Madathiparambil Saju, Kumar Nadimuthu, Rajani Sarojam

**Affiliations:** Temasek Life Sciences Laboratory, National University of Singapore, Singapore, Singapore

**Keywords:** RNAi, peltate glandular trichomes, Aux/IAA, ARF, auxin, spearmint

## Abstract

Phytohormone auxin controls various aspects of plant growth and development. The typical auxin signalling involves the degradation of canonical Aux/IAA proteins upon auxin perception releasing the auxin response factors (ARF) to activate auxin-regulated gene expression. Extensive research has been pursued in deciphering the role of canonical Aux/IAAs, however, the function of non-canonical *Aux/IAA* genes remains elusive. Here we identified a non-canonical *Aux/IAA* gene, *MsIAA32* from spearmint (*Mentha spicata*), which lacks the TIR1-binding domain and shows its involvement in the development of peltate glandular trichomes (PGT), which are the sites for production and storage of commercially important essential oils. Using yeast two-hybrid studies, two canonical Aux/IAAs, MsIAA3, MsIAA4 and an ARF, MsARF3 were identified as the preferred binding partners of MsIAA32. Expression of a R2R3-MYB gene *MsMYB36* and a cyclin gene *MsCycB2-4* was altered in *MsIAA32* suppressed plants indicating that these genes are possible downstream targets of MsIAA32 mediated signalling. Ectopic expression of *MsIAA32* in *Arabidopsis* affected non-glandular trichome formation along with other auxin related developmental traits. Our findings establish the role of non-canonical Aux/IAA mediated auxin signalling in PGT development and reveal species-specific functionalization of Aux/IAAs.

## 1 Introduction

Auxin plays a critical role in regulating multiple aspects of plant development and growth, leading to significant interest in understanding its signalling and response mechanism. The key players involved in auxin responsive transcription are transport inhibitor-resistant 1/auxin signalling F-box (TIR1/AFB) receptors, auxin/indole-3-acetic acid (Aux/IAA) repressors and auxin response factors (ARF) (Nemhauser, 2018). Canonical auxin signalling is based on TIR1/AFB mediated Aux/IAA protein ubiquitination and degradation upon auxin perception leading to ARF’s derepression and the activation of auxin-responsive gene regulation. All canonical Aux/IAAs possess four conserved domains (I–IV). The smallest Domain I has the “LxLxL” motif, known as the ethylene response factor (ERF)-associated amphiphilic repression (EAR) motif (Luo et al., 2018) which is important for conferring repression. Domain II has the “GWPPV” degron motif and controls the Aux/IAA proteins’ turnover by interacting with the E3 protein-ubiquitin ligase, SCF^TIR1^ (SKP1, Cullin, and TIR1 F-box-containing proteins) (Ramos et al., 2001). SCFs ubiquitinate their targets, marking them for degradation by the 26S proteasome (Deshaies, 1999). Domains III and IV together form type Bem1p (PB1) and I/II Phox domains that mediate hetero- and homodimerization of Aux/IAA proteins and heterodimerization of ARF and Aux/IAA proteins (Reed, 2001; Luo et al., 2018). Non-canonical Aux/IAAs are characterized by the absence of either full or partial domain I, II and/or III and are presumed to function differently from canonical Aux/IAAs. Two nuclear localization signals (NLSs) are found in canonical Aux/IAAs compared to only one NLS in non-canonical Aux/IAAs (Wu et al., 2017). Both canonical and non-canonical Aux/IAAs regulate gene expression by binding with other Aux/IAAs or ARFs. Similar to domains III and IV of Aux/IAA proteins, ARF proteins have a C-terminal dimerization domain (CTD (Li et al., 2016). They also have a well-preserved N-terminal region that binds DNA and specifically targets auxin response elements (AuxRE) in the promoter regions of genes regulated by auxin (Tiwari et al., 2003). These AuxRE sites contain variations of the TGTCNN motif, which are involved in the response to auxin in *Arabidopsis* (Zemlyanskaya et al., 2016). ARFs have a middle region that functions as a repression domain (RD) or an activation domain (AD) depending on the amino acid sequences present, and accordingly, they are called as activator ARFs or repressor ARFs (Li et al., 2016). Canonical auxin signalling is well studied and documented but with the emergence of non-canonical Aux/IAAs, information regarding their functions and signalling mechanisms remains limited. Only a few non-canonical *Aux/IAA* genes have been characterized till date. Study on two non-canonical *Aux/IAA* genes *IAA30* and *IAA20* in *Arabidopsis*, highlighted their importance in vascular pattering (Müller et al., 2016). Another study in rice showed the involvement of a non-canonical *Aux/IAA*, *OsIAA26* in ethylene regulated root growth (Chen et al., 2018). Recently the role of *IAA32* and *IAA34* in regulating differential growth of apical hook (Cao et al., 2019) and *IAA33* in maintaining root distal stem identity in *Arabidopsis* were established (Lv et al., 2020).

Glandular trichomes are specialized structures that produce and store a diverse array of specialized metabolites, which are critical for plants’ fitness and their interaction with the surrounding environment. The model plant *Arabidopsis* lacks such structures and possesses non-glandular trichomes. Spearmint possesses specialized structures called peltate glandular trichomes where valuable essential oils are produced (Reddy et al., 2017). These “green bio-factories” are located in the aerial tissues of aromatic plants, producing and storing large quantities of volatile metabolites (Turner et al., 2000; Champagne and Boutry, 2013; Lange and Turner, 2013). Since these metabolites have high commercial value, understanding the development of PGTs is of great interest. Studies in tomato and cotton have established the role of auxin as an important regulator of trichome development (Li et al., 2021) but minimal knowledge is available on the role of Aux/IAAs in glandular or non-glandular trichome development (Deng et al., 2012b; Zhang et al., 2015). The only well characterized *Aux/IAA* gene involved in trichome development is a canonical *Aux/IAA*, *SIIAA15* from tomato. Downregulation of *SIIAA15* which shows ubiquitous expression across many tissues resulted in multiple phenotypes along with reduced trichome number (Deng et al., 2012b). Later trichome enriched ARFs, *SlARF4* and *SIARF3* were identified from tomato which regulates auxin induced glandular and non-glandular trichome development (Zhang et al., 2015; Yuan et al., 2021). *SlARF4* was shown to mediate trichome development through MYBs and cyclin genes (Yuan et al., 2021). Recently *ARF1* was characterized from *Artemisia annua* as positive regulator of glandular trichome formation and artemisinin biosynthesis (Guo et al., 2023). But no information is available on the role of non-canonical Aux/IAAs in trichome development.

In this study, we isolated and characterized a novel non-canonical *Aux/IAA* gene, *MsIAA32*, from spearmint which regulates PGT development. Phylogenetic analysis showed that MsIAA32 belongs to the same clade of non-canonical Aux/IAA proteins as *Arabidopsis* IAA32 and IAA34 that lack domain II. Down-regulation of *MsIAA32* in spearmint lead to a decrease in the number of PGTs. Using yeast two-hybrid assay, MsARF3, MsIAA3 and MsIAA4 were identified as the binding partners of MsIAA32. The possible role of *MsIAA32* in non-glandular trichome formation was confirmed by ectopic expression in *Arabidopsis*. Along with an increase in trichome density, typical auxin-related phenotypes like reduced leaf size, decreased lateral roots and curled leaves, were also observed. Our results demonstrate the involvement of a non-canonical Aux/IAA protein in glandular and non-glandular trichome formation, and it also shows species-specific functionalization of Aux/IAAs. Further, identifying new gene targets controlling glandular trichome numbers in aromatic plants will provide new ways to increase secondary metabolite production and improve plant stress response.

## 2 Methods

### 2.1 Plant material, transformation, and selection of transgenics

Spearmint (*M. spicata*) and tobacco (*N. benthamiana)* were grown in a greenhouse under natural conditions. Columbia ecotype of *Arabidopsis thaliana* was used as the wild-type (WT). Plants were grown in a growth chamber maintained at 20°C with light conditions of 16 h light and 8 h dark. *Agrobacterium-*mediated transformation of spearmint was carried out using a previously published protocol (Reddy et al., 2017). Spearmint transgenics were screened for the fluorescence of the visual marker, GFP. Transformation of tobacco was pursued as described previously (Gallois and Marinho, 1995). Tobacco transgenics were screened for the fluorescence of their visual marker, mCherry. Genomic DNA (gDNA) was then extracted from GFP/mCherry-positive plants and used as a template for genotyping the transgenic lines. Southern blot analysis was pursued on the DNA-positive lines. The floral dip method was used for transforming *Arabidopsis* as described previously (Zhang et al., 2006). *Arabidopsis* lines were screened by basta selection (10 mg/L) and GFP fluorescence. T3 generation plants were used for analysis.

### 2.2 RNA isolation and quantitative real-time PCR

PGTs were isolated from 2-3 cm leaves as previously described (Champagne and Boutry, 2013). Later, total RNA was isolated from PGTs and other tissues. cDNA was synthesized from 500 ng of RNA and the gene expression levels in various tissues were analyzed by qRT-PCR. The expression was normalized using the GAPDH for spearmint and Tubulin for *Arabidopsis* and analyzed through the comparative ΔΔ^CT^ method (Livak and Schmittgen, 2001).

### 2.3 Gene amplification and plasmid construction

The sequences were amplified and cloned into pENTR™/D-TOPO^®^ gateway vector (Invitrogen, Germany). LR recombination transferred the cloned target gene into destination vectors, and the destination plasmids were transformed into Agrobacterium EHA105 by heat shock which was used for further experiments. The primer sequences are shown in **Supplementary Table S1**.

### 2.4 Subcellular localization and BiFC

The *MsIAA32* ORF was cloned into the pENTR/D-TOPO gateway vector (Invitrogen, Germany). *MsIAA32*/pENTR vector was then transferred into the pBA-DC-YFP destination vector (Zhang et al., 2005), which contains the C-terminal in frame with YFP and CaMV 35S promoter. For BiFC, donor vectors were transferred into pBA-^C^YFP-DC or pBA-^N^YFP-DC. *Agrobacterium* strain EHA105 was transformed with the constructs by heat shock. The EHA105 cells transformed with the constructs were cultured overnight at 28°C and 200 rpm, then resuspended in a solution containing 10 mM MES pH 5.6, 100 *µ*M acetosyringone and 10 mM MgCl_2_ to 1 OD_600_. After a 3 h incubation, the solutions were infiltrated in combinations of 1:1 ratio into one-month-old *N. benthamiana* leaves and fluorescence signals were viewed using confocal scanning laser microscope two days later.

### 2.5 Auxin treatment

For auxin-mediated PGT induction study, 2,4-D (10 mg/L, 0.045mM) was sprayed every two days for ten days on the top leaves of one-month-old WT spearmint plants. Newly grown leaves were subjected to SEM analysis. For auxin-mediated gene expression study, 100 mM IAA was sprayed onto leaves of one-month-old spearmint plants and the top two 2 cm leaves were collected at 0, 2, 5, 10, 30 and 60 min time points for RNA isolation and expression analysis.

### 2.6 Southern blot analysis

Southern blot was performed on DNA-positive lines. 15 µg of genomic DNA was cut with NdeI overnight and then run on a 1% (w/v) agarose gel at 40 V for 5 h. The gel was then washed with 250 mM HCL for 15 min with shaking at RT, followed by a wash with distilled water and soaked twice in denaturation solution (0.5 M NaOH and 1.5 M NaCl) for 15 min each at RT with gentle shaking. The gel was again washed with water and submerged twice in neutralization solution (0.5 M Tris-HCL and 1.5 M NaCl) for 15 min each at RT with gentle shaking. The gel was then transferred to nylon membrane by capillary transfer method. Next day, the wet membrane was UV-crosslinked using a crosslinker followed by a brief water rinse. The membrane was then hybridized with CaMV 35S promoter probe. Next day, the membrane was rinsed with 2x wash solution (2x saline-sodium citrate (SSC) and 0.1% SDS) for 5 min each at RT with gentle shaking. Later, the membrane was rinsed with 0.5x wash solution (0.5x SSC and 0.1% SDS) for 15 min each at 68°C with gentle shaking. The membrane was then submerged in washing buffer for 1 min and blocked by incubating in blocking buffer for 3 h at RT with gentle shaking. Later the membrane was soaked in fresh blocking solution containing anti-DIG antibody (150 U/ml) and incubated for 30 min at RT with gentle shaking. The membrane was then washed twice with washing buffer for 15 min each at RT with gentle shaking followed by equilibration in detection buffer for 2 min. The membrane was then placed in a clean, transparent sheet, and diluted CDP-star (1:300) was added on top of it. Finally, the number of T-DNA insertions were analyzed using a ChemiDoc imaging system (BioRad).

### 2.7 GC-MS analysis

For GC-MS analysis, 2-3 cm leaves (4-6) were ground to powder with liquid nitrogen and mixed with 500 µl ethyl acetate containing camphor (50 µg/ml) as an internal control. The samples were shaken for 10 minutes at room temperature, then centrifuged for 10 minutes at 13,000 rpm. The top organic layer was dehydrated with anhydrous Na_2_SO_4_ and analyzed using GC-MS (Agilent Technologies, USA). 2 µl of sample was injected and separated using a HP-5 MS column with a temperature program of 50°C for 1 min increasing at 8°C/min to 300°C and held for 5 min.

### 2.8 Promoter cloning and GUS staining

GenomeWalker™ Universal kit was used to amplify the flanking sequences of *MsIAA32.* The identified 729-bp promoter region of *MsIAA32*, was amplified and cloned into pENTR™/D-TOPO^®^ which was then transferred into pBGWFS7 by LR recombination and later used to transform *Agrobacterium* EHA105. For *MsIAA32* promoter analysis, transgenic tobacco lines were generated using the recombinant EHA105 Agrobacterium strain. The transgenic lines were tested for GUS staining by immersing the tissue in a solution (100mM sodium phosphate buffer (pH 7), 0.1% Triton X-100, 2 mM potassium ferrocyanide, 1 mg/ml 5-bromo-4-chloro-3-indolyl-β-D-glucuronide, 2 mM potassium ferricyanide and10 mM EDTA) and incubating in in the dark overnight at 37°C. Later, the tissue was cleared of chlorophyll by treating with 70% ethanol, and the stained GUS was captured using a Zeiss Whitefield microscope.

### 2.9 Yeast two-hybrid assay

Matchmaker Gold yeast two-hybrid library screening system was used to identify and confirm the binding partners of MsIAA32. ORF of *MsIAA32* was cloned into pGBKT7 vector and transformed into Y2H gold yeast, which was then used to screen a library generated by cloning spearmint leaf cDNA transcripts into pGADT7 vector. Positive clones were selected in SD/–Ade/–His/–Leu/–Trp/X-a-Gal/AbA plates and confirmed by colony PCR. ORFs of identified targets were individually cloned into pGADT7 vector and subjected to two-hybrid screening to confirm interaction. p53 bait and prey vectors provided in the kit were used as positive controls. Yeast transformation was implemented as mentioned in the user manual of Matchmaker Gold Yeast two-hybrid system.

### 2.10 Scanning electron microscope

For Scanning Electron Microscopy (SEM), similar sized leaves from WT and selected RNAi lines were collected from the same node and observed under a scanning electron microscope (Joel). One leaf each from three different plants of each line was selected and eight identical spots of same diameter were imaged for each selected leaf of WT and RNAi lines to avoid variation. All 24 images from each line were used for counting the number of trichomes. For epidermal cells and stomatal counting, identical spots from three similar sized leaves were imaged. For *Arabidopsis* overexpression lines and WT, eight identical spots of same diameter from leaf number six were chosen (n=3, 24 images per line). The number of trichomes, epidermal cells and stomata were measured using ImageJ. Trichome density assessment was done as described previously with slight modifications (Watts and Kariyat, 2021). Images were captured at 50x magnification for spearmint which contains ~ 4.78 mm^2^ leaf area and 25x for *Arabidopsis* which contains ~19.38 mm^2^ leaf area as measured by Image J. Trichome density per mm^2^ was calculated by dividing the number of trichomes in 50x magnified image by 4.78 for spearmint and by dividing the number of trichomes in 25x magnified image by 19.38. Similarly, epidermal cells and stomatal density per mm^2^ was calculated by dividing the number of epidermal cells and stomata in 900x and 350x magnified images respectively by their corresponding leaf areas of ~ 0.014 mm^2^ and 0.104 mm^2^.

### 2.11 Phylogenetic analysis

MEGA11 was used to construct the phylogenetic tree using Maximum Likelihood method with bootstrap values of 1000 replicates. *A. thaliana* and *Solanum lycopersicum* sequences were taken from NCBI database.

### 2.12 Statistical analysis

The data is shown as “mean ± standard deviation (SD)” based on 3 to 6 biological replicates, each with 3 duplicates. Significance between transgenic plants and WT was evaluated using a two-tailed Student’s t-test and is indicated by asterisks. Asterisk notation: * signifies p < 0.05; ** signifies p < 0.01; *** signifies p < 0.001.

## 3 Results

### 3.1 Identification and characterization of a PGT-specific non-canonical *MsIAA32*

**Figure 1:**
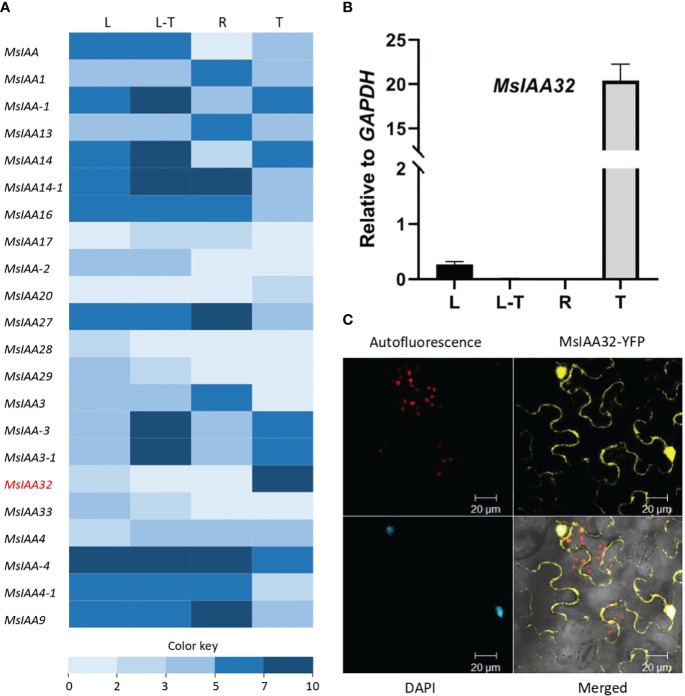
Expression analysis of *MsIAA32*. **(A)** A heat map of spearmint *Aux/IAAs* expression in different tissues: PGTs (T), leaf stripped of PGTs (L-T), leaf (L) and roots (R). *MsIAA32* is shown in red and demonstrates high expression in PGTs. **Supplementary Table S2** has expression values and sequence data. **(B)** *MsIAA32* shows specific expression in PGTs. **(C)** *N. benthamiana* leaf cells showing nucleus and cytoplasm localization of MsIAA32.

From the previously generated transcriptome data of four different tissues of spearmint, namely leaves devoid of PGTs, PGTs, leaves and roots (Jin et al., 2014), 22 *Aux/IAAs* were annotated (**Figure 1A**). Out of these *Aux/IAAs*, *MsIAA32*, showed PGT-specific expression and was selected for further characterization. This was validated by qRT-PCR across various tissues (**Figure 1B**). The full-length ORF of *MsIAA32*, consisting of 540 bp and encoding a 180 amino acid polypeptide, was amplified from PGT cDNA. MsIAA32 showed the highest sequence similarity (~76%) to *Sesamum indicum* IAA32 in NCBI protein BLAST analysis. The amino acid sequences of known *Solanum lycopersicum* (Sl) IAAs and *Arabidopsis thaliana* (At) IAAs were used to construct a phylogenetic tree (**Supplementary Figure S1**). Previous phylogenetic analysis of AtIAAs and SlIAAs has led the IAAs to be grouped into 11 distinct clades (A-K), of which Clade H, Clade I and Clade K members are considered as the non-canonical IAAs due to the absence of one or two of the conserved domains (Audran-Delalande et al., 2012). MsIAA32 fell under clade I along with AtIAA32, AtIAA34 and SlIAA32. A clustal alignment of MsIAA32 with IAA32 from other plants revealed the absence of domain II which is also observed in the members of Clade I, SlIAA32 (Audran-Delalande et al., 2012), AtIAA32 and AtIAA34 (Shimizu-Mitao and Kakimoto, 2014) (**Supplementary Figure S2**). Sequence of MsIAA32 was ~ 62%, 46% and 45% identical to the sequences of members of clade I, SlIAA32, AtIAA32 and AtIAA34 respectively. Additionally, a SV40-like nucleus localization signal (NLS) was present in domain IV (**Supplementary Figure S2**).

The subcellular localization of MsIAA32 was examined by fusing its ORF with a yellow fluorescent protein (YFP) and expressing it in *Nicotiana benthamiana* leaves using Agrobacterium infiltration under control of the CaMV 35S promoter. The recombinant protein was found to be localized in both nucleus and cytoplasm, as seen in **Figure 1C**. Such extranuclear localization is also observed for SlIAA32 (Audran-Delalande et al., 2012).

### 3.2 *MsIAA32* promoter shows trichome specific expression

**Figure 2:**
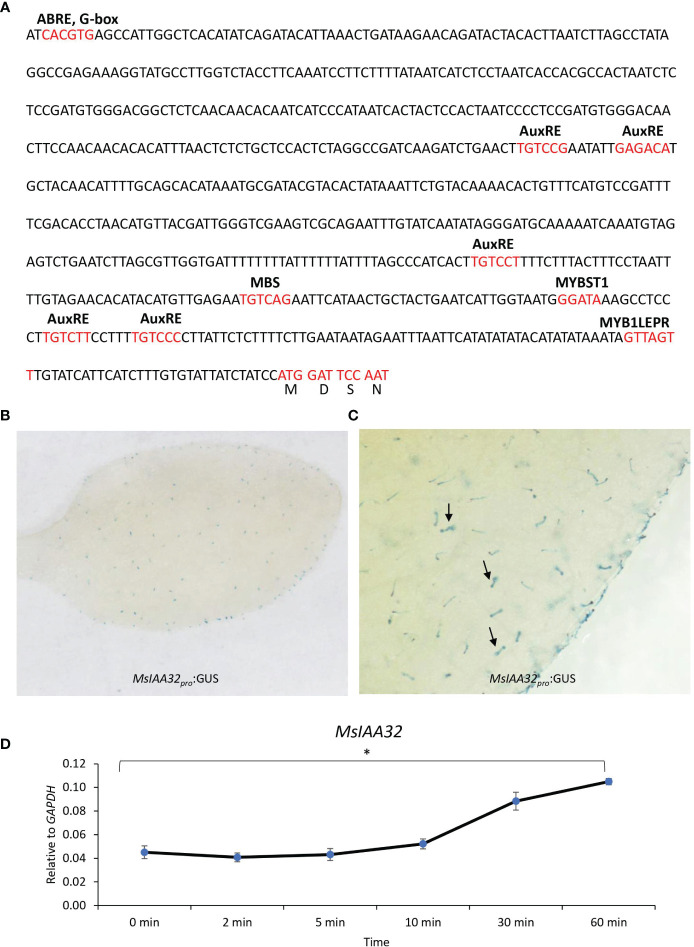
*MsIAA32* promoter analysis. **(A)** Promoter region of *MsIAA32* contains regulatory elements. **(B, C)** *MsIAA32pro*:GUS exhibit trichome-specific expression in *N. benthamiana* leaves, as indicated by black arrows pointing to GUS-stained trichomes. **(D)** qRT-PCR showing enhanced expression of *MsIAA32* after auxin treatment. *P < 0.05.

A 729-bp upstream DNA fragment of the translation start site was obtained via genome walking. PlantCARE (http://bioinformatics.psb.ugent.be/webtools/plantcare/html/) identified cis-acting regulatory elements in the promoter, including an AuxRE-motif, an auxin-responsive element (Ulmasov et al., 1995). Two variants of the core AuxRE (TGTC) sites with a 5-bp spacer (TGTCTT—–TGTCCC) were present (**Figure 2A**). ARFs are known to bind as dimers on palindromic AuxREs, which are spaced by 5 to 9 nucleotides (Michal et al., 2019) suggesting a probable regulation of *MsIAA32* by ARFs. Additionally, several TFs and light-responsive elements were also present. The expression pattern of the promoter was studied by pairing it with a β-glucuronidase (GUS) reporter gene and transforming it into *N. benthamiana* plants. Transgenic tobacco leaves displayed trichome-specific staining (**Figures 2B, C**).

### 3.3 MsIAA32 expression is induced by auxin

Presence of AuxRE sites in the promoter of *MsIAA32* indicates its possible regulation by auxin. Many *Aux/IAA* gene members are known to be induced by auxin and show the presence of AuxRE sites in its promoter. To test whether *MsIAA32* expression is induced by auxin, 100 mM of 3-Indoleacetic acid was sprayed to one-month-old WT spearmint plants and the expression of *MsIAA32* was analyzed at various time points as previously described (Wu et al., 2012). Increased expression of *MsIAA32* was noted 30 min after auxin treatment (**Figure 2D**).

### 3.4 Silencing of *MsIAA32* reduces the number of PGTs formed and monoterpene production in spearmint

**Figure 3:**
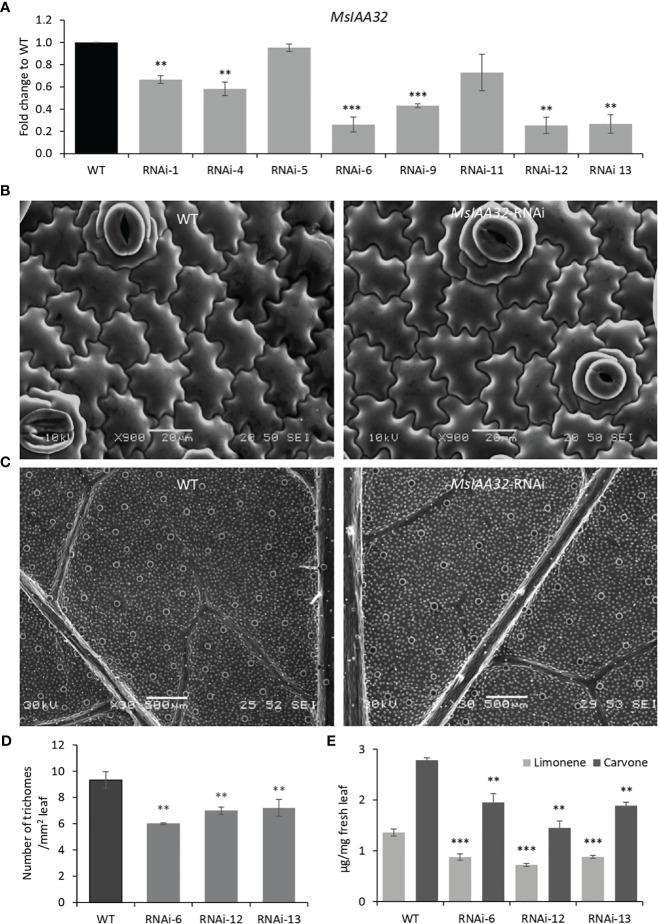
Characterization of *MsIAA32*-RNAi lines. **(A)** Reduced levels of *MsIAA32* in *MsIAA32*-RNAi lines. **(B)** SEM images of spearmint leaf showing similar epidermal cell size and number in WT and *MsIAA32*-RNAi line. **(C)** SEM images of spearmint leaf showing lesser PGTs (globular structures) in *MsIAA32*-RNAi line than the WT. Reduced number of PGTs **(D)** and reduced levels of carvone and limonene **(E)** in transgenic spearmint *MsIAA32*-RNAi lines compared to WT. **P < 0.01; ***P < 0.001

To study the function of *MsIAA32*, we created transgenic spearmint lines with an RNAi construct controlled by the CaMV 35S promoter and targeting *MsIAA32*. Thirteen transgenic lines were developed, which were confirmed by the southern blot, of which we selected eight independent single-copy lines for further analysis (**Supplementary Figure S3A**). qRT-PCR analysis showed that of the eight chosen lines, six lines displayed reduced levels of *MsIAA32* transcripts when compared to wild type (WT) (**Figure 3A**). Three best lines (RNAi-6, RNAi-12 and RNAi-13) were chosen for further characterization. The *MsIAA32* RNAi lines had a similar appearance to WT (**Supplementary Figure S3B**). No change in leaf size or shape was observed (**Supplementary Figure S3C**). A closer examination of leaf cells and PGTs in these plants was conducted using scanning electron microscopy. The epidermal cells and stomata did not show any significant changes in morphology or number (**Figure 3B**, **Supplementary Figures S3H, I**). Compared to WT plants *MsIAA32* RNAi plants had a reduced number of PGTs, but PGT morphology was not affected (**Supplementary Figures S3D-G**). In WT plants the density of PGTs ranges from 8 to 10 per mm^2^ while density of PGTs in *MsIAA32* RNAi was 6 to 7.5 per mm^2^. Overall ~ 22-35% reduction in trichome density was observed in *MsIAA32* RNAi lines compared to WT (**Figures 3C, D**).

In spearmint, PGTs are the site of production and storage of secondary metabolites, primarily monoterpenes (limonene and carvone) (Reddy et al., 2017). To assess any changes in volatile production in *MsIAA32*-RNAi transgenics, a gas chromatography-mass spectrometry (GC-MS) analysis was done, which revealed ~30-40% reduction in limonene and carvone content (**Figure 3E**). To investigate if downregulation of *MsIAA32* has any effect on the expression levels of the biosynthetic enzymes of the 2-*C*-methyl-D-erythritol 4-phosphate (MEP) pathway, we did a qRT-PCR of major biosynthetic enzymes of the MEP pathway. As shown in **Supplementary Figure S4**, the expression of enzymes remains unaltered. This suggests that the observed reduction in secondary metabolites in *MsIAA32* RNAi transgenics is mainly due to a decrease in the number of PGTs.

### 3.5 MsIAA32 binds with MsIAA3, MsIAA4 and MsARF3

**Figure 4:**
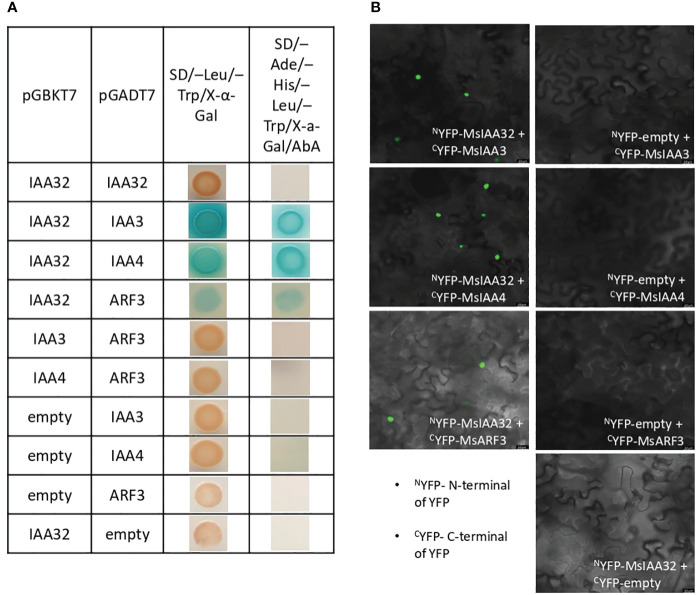
Interaction study between MsIAA32 and its partners. **(A)** Yeast two-hybrid assay between MsIAA32 and its partners. First low stringent screening was performed on SD/–Trp/–Leu/X-α-Gal selection medium where positive interactions should result in blue colonies. The positive interactions were further confirmed by growing on SD/–His/–Ade/–Trp/–Leu/AbA/X- α -Gal selection medium for more stringent screening. SD/–Trp/–Leu/X-α-Gal, SD medium without Leu and Trp but containing X-α-Gal (40 µg/ml); SD/–His/–Ade/–Trp/–Leu/AbA/X- α -Gal, SD medium without His, Ade, Trp and Leu but containing aureobasidin A (800 ng/ml) and X-α-Gal (40 µg/ml). **(B)** BiFC analysis of the interaction between MsIAA32 and its partners. The BiFC assays were conducted using the split YFP system. Tobacco epidermal cells were transformed with the different combinations of plasmids, and the YFP signals in the nucleus were observed with a confocal microscope. Scale bars, 20 µm. ^C^YFP, C-terminal of YFP; ^N^YFP, N-terminal of YFP.

Aux/IAA proteins are known to form homodimers or heterodimers with other Aux/IAAs or ARFs to regulate downstream genes (Piya et al., 2014). To identify the binding partners of MsIAA32, we did a yeast two-hybrid (Y2H) screen where MsIAA32 was used as a bait against the library of clones from spearmint leaf cDNA. Matchmaker Gold Yeast Two-Hybrid System was used for Y2H screen where bait and prey interactions activate transcription of four independent reporter genes (AUR1-C, ADE2, HIS3, and MEL1). MEL-1 encodes a-galactosidase, AUR1-C expression confers strong resistance to antibiotic Aureobasidin A, ADE2 and HIS3 provides the strain the ability to synthesis adenine and histidine respectively. From the list of partners, we identified two Aux/IAAs and one ARF as interacting partners of MsIAA32. Through NCBI blast, we identified and named the two IAAs as MsIAA3 and MsIAA4 and the ARF as MsARF3. All the three identified partners had expressions in PGTs as seen by their FPKM values within the various tissues of spearmint in RNA-Seq data (**Supplementary Figure S5**). To further confirm positive interaction between the MsIAA32 and its identified partners, individual Y2H assays between MsIAA32 and its partners were pursued which showed strong interaction between MsIAA32 and MsIAA3, MsIAA32 and MsIAA4 and weak interaction between MsIAA32 and MsARF3 (**Figure 4A**). MsIAA3 and MsIAA4 did not interact with MsARF3. All the positive interactions were further confirmed using Biomolecular fluorescence complementation (BiFC) assay. MsIAA32 fused to ^N^YFP and MsIAA3/MsIAA4/MsARF3 fused to ^C^YFP showed a strong fluorescence signal in the nucleus (**Figure 4B**). However, no signal was observed in the empty vectors.

MsIAA3 and MsIAA4 sequences had all the conserved domains (I-IV) of a canonical IAA (**Supplementary Figure S6**). A phylogenetic analysis of MsIAA3 and MsIAA4 with all other IAAs from *Arabidopsis thaliana* and *Solanum lycopersicum* placed MsIAA3 and MsIAA4 under clade A (**Supplementary Figure S1**). Gain-of-function mutations in AtIAA3 is known to cause short hypocotyls, altered auxin-regulated root development, and enlarged cotyledons (Tian et al., 2002). Recently it was shown that AtIAA3-mediated repression of PHYTOCHROME-INTERACTING FACTORs coordinates light and auxin signalling (Xi et al., 2021). Mutant studies of SlIAA4 has not been pursued, however expression analysis shows it has high expression in fruits and leaves (Audran-Delalande et al., 2012). This highlights that the function of MsIAA3 and MsIAA4 in glandular trichomes development needs to be deciphered.

A phylogenetic tree of MsARF3 with other AtARFs and SlARFs placed MsARF3 with a clade containing SlARF9, SlARF12, SlARF18, AtARF9, AtARF11 and AtARF18 (**Supplementary Figure S7A**), which are members of class 1a ARFs. This set of ARFs is majorly known to act as negative regulators (Xu et al., 2016). MsARF3 contains all the characteristic domains of an ARF, which are the B3 domain, ARF domain and Aux/IAA domains (**Supplementary Figure S7B**). In addition, it has a serine-rich middle region which is characteristic of a repressor ARF (Tiwari et al., 2003; Roosjen et al., 2018).

### 3.6 Auxin spray can induce PGT formation

**Figure 5:**
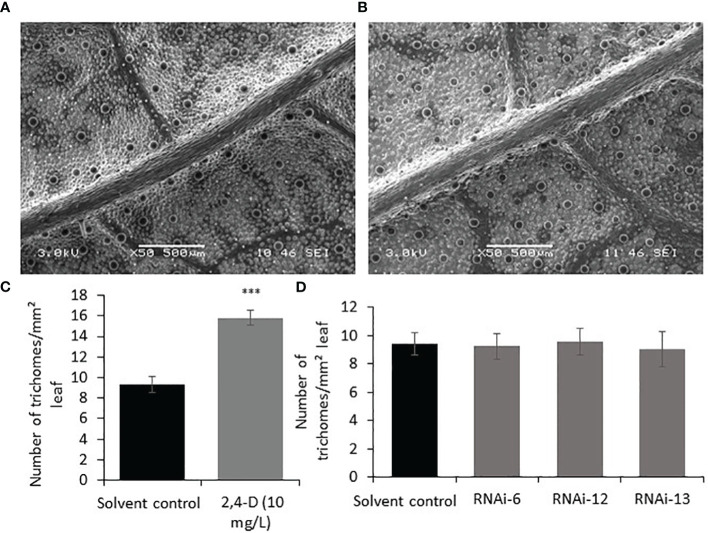
Effect of auxin on PGT development and *MsIAA32* expression. **(A)** SEM image of spearmint leaf treated with water **(B)** SEM image of spearmint leaf treated with auxin (10 mg/L 2,4-D, 0.045mM) showing more PGTs. **(C)** Graph depicting the increased number of PGTs in auxin treated leaves compared to solvent treated leaves. **(D)** Graph depicting the recovery of PGT number in *MsIAA32* RNAi lines treated with auxin (10 mg/L 2,4-D, 0.045mM) to levels observed in solvent treated WT leaves. ***P < 0.001.

To investigate the effect of auxin on PGT development, 2,4-Dichlorophenoxyacetic acid (2,4-D) (10 mg/L, 0.045mM) was sprayed every two days for ten days on the apical regions of one-month-old WT spearmint plants. Newly grown leaves were then subjected to SEM analysis and trichome density analysis. We found ~ 60% increase in the number of PGTs in auxin treated leaves when compared to solvent treated leaves (**Figures 5A–C**). A similar result was also observed previously in tomato plants (Yuan et al., 2021). To examine if auxin spray can rescue the *MsIAA32* RNAi phenotype, a similar spray experiment was performed as mentioned above. Auxin treatment led to the restoration of PGT density to wild type levels in the *MsIAA32* RNAi plants (**Figure 5D**).

### 3.7 Expression of a *R2R3-MYB* and a B2-type cyclin gene is altered in *MsIAA32* down-regulated lines

**Figure 6:**
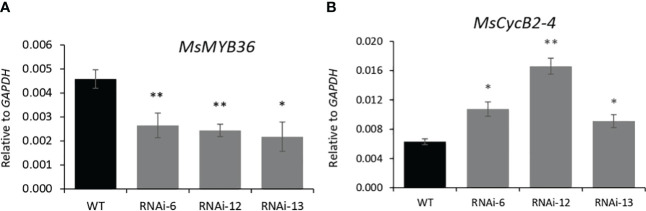
Characterization of *MsMYB36* and *MsCycB2-4.* Reduced levels of *MsMYB36* **(A)** and increased levels of *MsCycB2-4* **(B)** in *MsIAA32*-RNAi lines. *P < 0.05; **P < 0.01.

MYB genes are known to regulate trichome development in various plants, and in tomato, trichome enriched MYBs mediate auxin dependent formation of glandular and non-glandular trichomes (Yuan et al., 2021). To elucidate how *MsIAA32* regulates PGT development in spearmint, we mined a list of potential MYB candidates from our RNA-Seq data which had high expression in PGTs. Expression of these MYB genes were analyzed in *MsIAA32* RNAi lines, using qRT-PCR. One R2R3-MYB, *MsMYB36*, was significantly reduced in RNAi plants compared to WT plants (**Figure 6A**). Sequence analysis showed the presence of conserved features of R2R3-MYBs, a R2, R3 repeat and five tryptophan residues within the R2 and R3 repeats, which together forms a helix-turn-helix motif at the N-terminus (Dubos et al., 2010) (**Supplementary Figure S8A**). *Arabidopsis* MYBs were used to construct a phylogenetic tree (**Supplementary Figure S8B**). MsMYB36 fell under the S14 subfamily, members of which have been reported to function in the regulation of axillary meristems and root development (Keller et al., 2006; Müller et al., 2006).

In addition, expression of B2-type cyclins genes was also investigated as recently, a tomato cyclin, *SlCycB2*, and tobacco cyclin, *NtCycB2* were identified to function as inhibitors of glandular and non-glandular trichome formation (Yuan et al., 2021; Zhang et al., 2021; Wang et al., 2022). Additionally, tomato cyclin, *SlCycB2* was also shown to be regulated by MYBs involved in trichome formation. We screened the B2-type cyclins from our RNA-Seq data and found one cyclin, *MsCycB2-4*, which showed expression in PGTs, to have enhanced expression in the *MsIAA32*-RNAi line compared to WT by qRT-PCR (**Figure 6B**). The overexpression of *MsCycB2-4* might be the reason for the decrease in PGT numbers in *MsIAA32* RNAi lines.

### 3.8 Ectopic expression of *MsIAA32* causes diverse phenotypes in *Arabidopsis*

**Figure 7:**
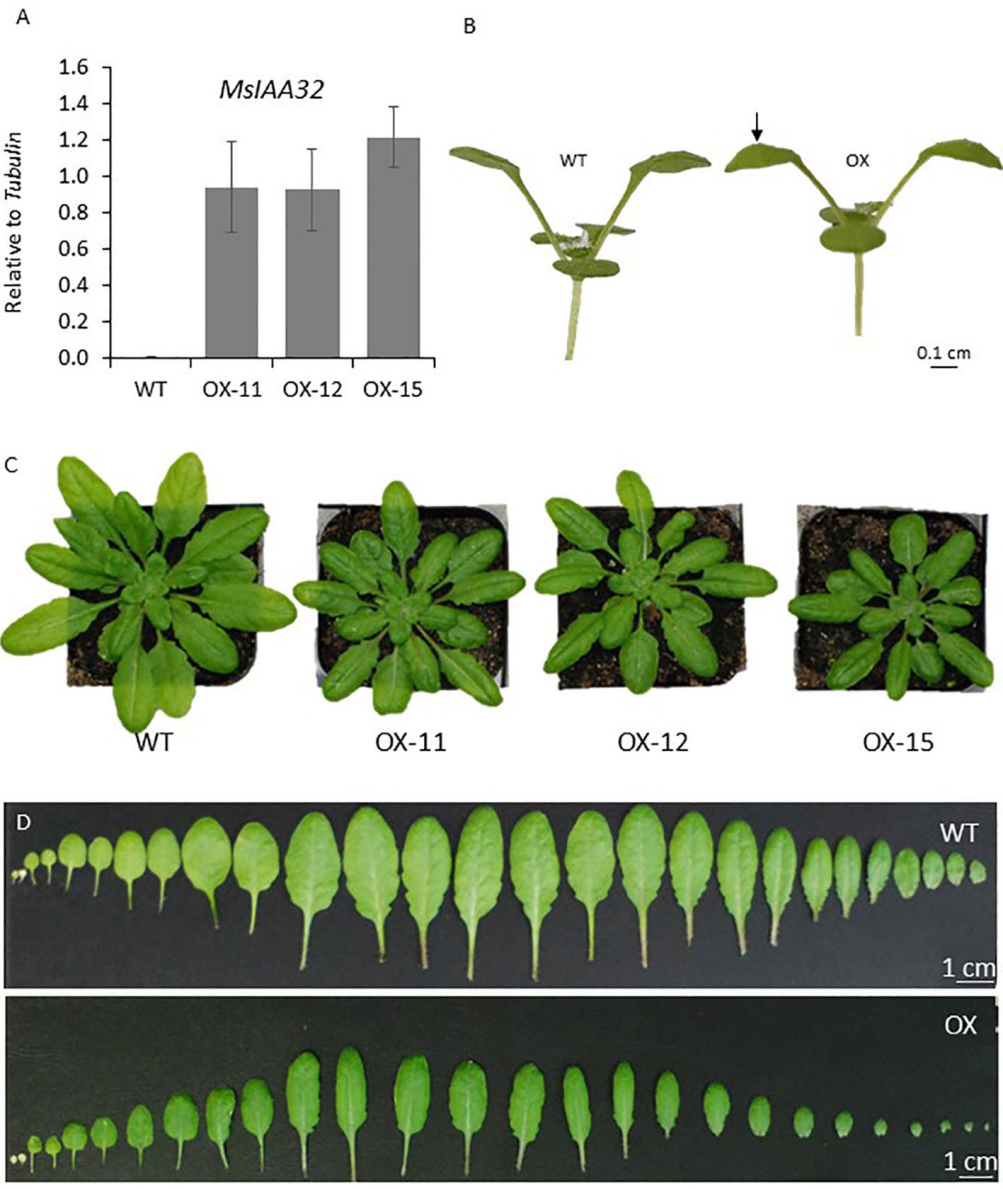
Characterization of *Arabidopsis* plants expressing *MsIAA32*. **(A)** Ectopic expression of *MsIAA32* in *Arabidopsis* plants. **(B)** *MsIAA32* expressing *Arabidopsis* plant showing epinasty. The black arrow denotes the downward curling of the leaf. **(C)** *MsIAA32* expressing *Arabidopsis* plants are visibly smaller in size when compared to WT. **(D)** Cotyledons and leaves excised from WT and *MsIAA32* expressing *Arabidopsis*. The two cotyledons are shown on the left, followed by the vegetative leaves in the order they initiated. WT, Wildtype; OX, *MsIAA32* expressing *Arabidopsis* line.

To observe if *MsIAA32* has an influence on non-glandular trichome development, it was ectopically expressed using CaMV35S promoter in *Arabidopsis*. We generated multiple transgenic lines and selected the three with the strongest phenotype for further study. *MsIAA32* expression was confirmed by qRT-PCR in these lines (**Figure 7A**). The transgenic plants exhibited several auxin-related phenotypes. The leaves exhibited inward curling (**Figure 7B**). The leaves and the rosette diameter were smaller (**Figures 7C, D**).

**Figure 8:**
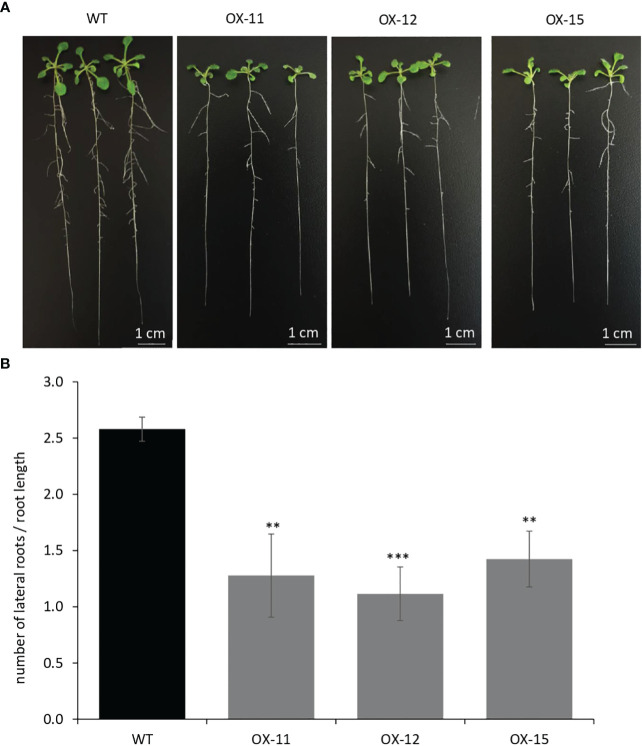
Quantification of lateral roots in *MsIAA32* expressing *Arabidopsis* plants. **(A, B)** *MsIAA32* expressing *Arabidopsis* plants showing reduced lateral roots when compared to plants 12 days after germination. WT, Wildtype; OX, *MsIAA32* expressing *Arabidopsis* line. **P < 0.01; ***P < 0.001

**Figure 9:**
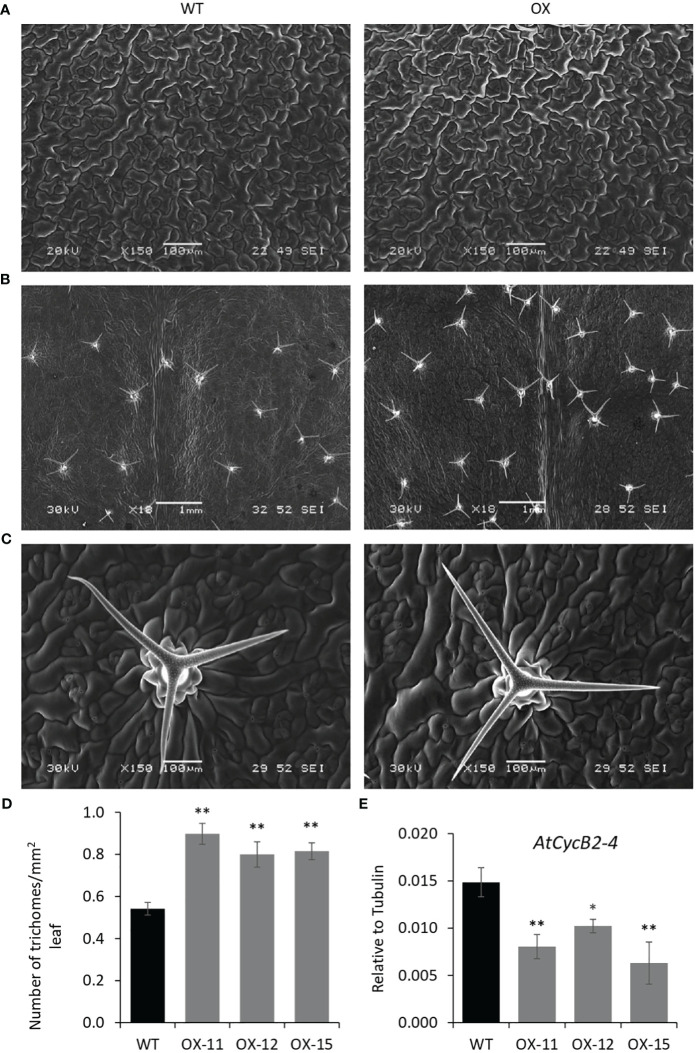
Quantification of trichomes in *MsIAA32* expressing *Arabidopsis* plants. **(A)** SEM images of *Arabidopsis* leaves showing similar size and number of epidermal cells. **(B)** SEM images of *Arabidopsis* leaves showing an increased number of trichomes. **(C)** SEM image of WT leaf and *MsIAA32* expressing leaf showing similar PGT shape and size. **(D)** Increased number of trichomes in *MsIAA32* expressing *Arabidopsis* plants compared to WT. **(E)** Reduced expression of *AtCycB2-4* in *MsIAA32* expressing *Arabidopsis* plants. WT, Wildtype; OX, *MsIAA32* expressing *Arabidopsis* line. *P < 0.05; **P < 0.01.

The seedlings were smaller with reduced lateral roots (**Figures 8A, B**). Phenotypically adult transgenic plants looked similar in stature to WT plants. A closer examination of leaf cells and PGTs in these plants was conducted using scanning electron microscopy. The epidermal cells had no change in size or number (**Figure 9A**), however, the trichome density was increased by ~ 60% in the transgenic lines, with no effect on the size and shape of trichomes (**Figures 9B–D**). To test whether any B-type cyclin is involved in the trichome phenotype observed in *Arabidopsis* transgenics, the ortholog of *MsCycB2-4* was identified, and the expression analysis was performed using q-RT PCR. As expected, *AtCycB2-4* (NM_106281.6) had reduced expression in *Arabidopsis* transgenics when compared to WT, which might be a possible reason for the increase in the number of trichomes in transgenics since CycB2-4 appears to be a negative regulator of trichome initiation (**Figure 9E**) (Yuan et al., 2021; Zhang et al., 2021; Wang et al., 2022).

## 4 Discussion

The role of canonical Aux/IAAs in plant development and growth through auxin-regulated gene expression is well studied. However, with the identification of non-canonical Aux/IAAs that lack one or more of the four conserved domains, the presence of an alternate non-canonical auxin signalling pathway is now established. The majority of our knowledge in understanding the functions of *Aux/IAA* genes in plant growth and development comes from research on *Arabidopsis*, revealing extensive overlap in function among its *Aux/IAA* gene family. However, studies in Solanaceae demonstrate specialized functions of these genes, highlighting the need to study them in diverse plants for a deeper understanding of their significance. *Arabidopsis* contain six non-canonical *Aux/IAA* genes (*IAA34*, *IAA33*, *IAA32*, *IAA31*, *IAA30*, and *IAA20*), while tomato has only two non-canonical *Aux/IAA* genes (*Sl-IAA33* and *Sl-IAA32*), which lack typical domains I and II (Audran-Delalande et al., 2012). In this study, we identified a non-canonical *Aux/IAA* gene *MsIAA32* from spearmint involved in PGT development. Phylogenetic analysis puts MsIAA32 in the same clade as Sl-IAA32 and AtIAA32, with which it exhibits 62% and 46% sequence similarity. *SlIAA32* mutants have not been characterized, but its expression is limited to fruits and is shown to function as a repressor of auxin signalling (Audran-Delalande et al., 2012). Single mutants of *AtIAA32* show no obvious phenotype, but along with *At1AA34*, it redundantly controls apical hook maintenance (Cao et al., 2019). Compared to canonical auxin signalling, insights into the mechanisms of non-canonical Aux/IAA mediated auxin signalling remains limited.

In typical canonical auxin signaling, auxin induces degradation of Aux/IAAs protein, thus releasing the inhibition of ARF transcription factors, leading to transcriptional reprogramming of auxin responsive genes. Interestingly many Aux/IAAs possess AuxRE binding sites to which ARFs bind, hence their transcription is concomitantly upregulated by auxin, thus establishing a negative feedback loop to generate an appropriate auxin response (Guilfoyle and Hagen, 2007). In contrast, studies in non-canonical Aux/IAAs, AtIAA32, AtIAA33, and AtIAA34 have shown that auxin promotes their protein accumulation through phosphorylation which increases their stability (Cao et al., 2019). It was also shown that auxin treatment had no effect on the transcription of *AtIAA33* (Lv et al., 2020). However expression of non-canonical *Aux/IAAs* like *AtIAA20* and *AtIAA30* were shown to be induced by auxin (Sato and Yamamoto, 2008). But increased levels of auxin did not have any effect on IAA20 protein longevity (Dreher et al., 2006). In summary, unlike canonical Aux/IAAs where auxin mediates the destruction of their proteins and generally upregulates their transcription, non-canonical Aux/IAAs response to auxin can vary at transcriptional and protein level. Auxin treatment was able to increase the expression of *MsIAA32* but the influence of auxin on MsIAA32 protein needs to be investigated. Lack of domain II should render MsIAA32 protein insensitive to auxin mediated degradation hence their levels should probably remain unchanged under high auxin. In our study, auxin treatment was able to rescue the *MsIAA32* RNAi phenotype. This might be probably due to the increased transcription resulting in increased protein levels in RNAi plants leading to PGT number restoration. Further studies need to be performed with regards to MsIAA32 steady state levels and influence of auxin on its protein stability to better understand the role of *MsIAA32* in auxin mediated PGT development.

Generally, Aux/IAAs are known to possess two kinds of putative nuclear localization signals, with a basic residue-rich SV40-type NLS in domain IV and a bipartite structure between domains I and II with a conserved basic doublet “KR” (Ke et al., 2019). However, MsIAA32 only has the SV40-type NLS and lacks the bipartite NLS, which might be the reason for its extended localization in the cytosol in addition to the nucleus. The lack of Domain II, which is involved in protein degradation, may also contribute towards the extended localization. Similar subcellular localization to nucleus and cytoplasm was also observed for SlIAA32, AtIAA32, and AtIAA34 (Audran-Delalande et al., 2012; Cao et al., 2019). The nuclear localization indicates a transcriptional regulatory function of MsIAA32, and the presence of MsIAA32 in cytoplasm suggests probable novel interactions with binding factors specifically available in the cytoplasm.

From yeast two-hybrid studies, it was evident that MsIAA32 prefers binding with MsIAA3 or MsIAA4 (**Figure 4A**), both of which formed a clade with canonical AtIAA3 in phylogenetic analysis (**Supplementary Figure S1**). AtIAA3 was previously identified to bind with activator ARFs (Piya et al., 2014) and is involved in light and auxin signalling in *Arabidopsis* (Xi et al., 2021). Aux/IAA proteins are known to interact with other Aux/IAA proteins but the biological significance of these interactions in auxin signalling has not been well elucidated. In *Arabidopsis*, an interactome map of all the 29 Aux/IAA proteins was constructed, which shows that the 29 *Aux/IAA* genes could interact with each other via 253 interactions (Luo et al., 2018). Given that MsIAA32 is a non-canonical Aux/IAA hence more stable, these types of interactions among canonical and non-canonical Aux/IAA proteins probably add more flexibility in fine tuning the activity of the auxin signalling network. Further research is required to explore the biological significance of interactions between non-canonical MsIAA32 and canonical MsIAA3/MsIAA4 towards PGT development. MsIAA32 was also able to bind with MsARF3. ARFs play a crucial role in defining auxin response specificity by regulating the expression of transcription factors that depend on auxin. ARFs are divided into three conserved classes: A, B, and C. Class A ARFs act as transcriptional activators, while class B and C ARFs function as transcriptional repressors (Chung et al., 2019). MsARF3 is a class B repressor ARF which is supported by its serine-rich middle region, a characteristic feature of repressor ARFs (Roosjen et al., 2018) (**Supplementary Figure S7B**).

Studies in Tomato have revealed the importance of auxin mediated signalling in trichome formation. Tomato leaves possess eight different types of non-glandular and glandular trichomes (McDowell et al., 2010). In tomato, two ARFs have been characterized which are involved in trichome formation in leaves, SlARF3 was found to positively regulate the density of glandular type I and VI and non-glandular type V trichomes (Zhang et al., 2015), and SlARF4 was shown to positively regulate the auxin-induced formation of II, V and VI type trichomes. It was determined that SlARF4 regulates trichome development through repressing two R2R3-MYB genes, *SlTHM1* and *SlMYB52*. These MYB genes, in turn, controlled trichome formation through the regulation of the B-type cyclin gene *SlCycB2* (Yuan et al., 2021). Studies in cell cycle genes, including cyclins in tomato and *Arabidopsis*, have shown that the transition from the mitotic phase to the endoreduplication cycle is key for the development of both unicellular and multicellular trichomes (Fambrini and Pugliesi, 2019). In tomato *SlCycB2*, is highly expressed in the trichomes and negatively regulates the formation of unicellular and multicellular trichomes. Recently SlMYB75 was identified to regulate trichome formation in tomato where SlMYB75 directly targeted the promoters of *SlCycB2*, *SlTHM1* and *SlMYB52* (Gong et al., 2021). Further, it was found that SlARF4 interacts with *SlMYB75* promoter and inhibits its expression. Taken together, it was proposed that in tomato, a *SlARF/SlMYB/SlCycB* transcriptional cascade controls the formation of type II, V, and VI trichomes. Regulation of these ARFs by Aux/IAAs is not known in tomato. The only other characterized *Aux/IAA* gene involved in trichome development is the canonical *Aux/IAA* gene *SlIAA15* from tomato. The down regulation of *SlIAA15* affected the development of both non-glandular and glandular types of trichomes. Given its ubiquitous expression pattern, downregulation of *SlIAA15* also showed diverse phenotypes like altered stem xylem development, thickened leaves, decreased fruit set, increased lateral root and reduced apical dominance revealing its multiple functions in plant development (Deng et al., 2012a; Deng et al., 2012b). Interestingly, the down regulation of *SlIAA15* resulted in the reduction in expression of few R2R3-MYB genes including *SlTHM1*gene. It was proposed that *SlIAA15* regulates trichome formation via direct or indirect control of MYB genes. Later in a different study looking into tomato fruit development showed that SlARF4, can interact with SlIAA15 in a BiFC assay (Shinozaki et al., 2018). Similar to the studies in tomato, MsIAA32 downregulation results in misexpression of a R2R3-MYB gene *MsMYB36* and B-type cyclin gene *MsCycB2-4* suggesting their involvement in PGT development. The exact mechanism of MsIAA32 mediated regulation of the possible *ARF/MYB/CycB* transcriptional cascade in spearmint towards PGT development through its binding partners needs further exploration. Role of *MsIAA32* in glandular trichome development highlights species-specific distinct and novel functions of Aux/IAA family members sustained by cell/tissue-specific expression patterns.

Apart from increased trichome number, overexpression of *MsIAA32* in Arabidopsis resulted in developmental phenotypes like downward leaf curling, reduced leaf size, smaller rosette diameter and decreased lateral root growth. These phenotypes have been associated with alteration in auxin homeostasis and signaling including misexpression of *Aux/IAA* genes (Rogg et al., 2001; Sato and Yamamoto, 2008; Arase et al., 2012; Rinaldi et al., 2012; Guseman et al., 2015; Liu et al., 2015; Sandalio et al., 2016; Xiong and Jiao, 2019). Recently overexpression of a non-canonical *Aux/IAA* gene *EgrIAA20* from *Eucalyptus* in *Arabidopsis* showed leaf curling, reduced leaf size and reduced diameter of rosette similar to *MsIAA32* along with other phenotypes (Yu et al., 2022). *Arabidopsis Aux/IAA* genes show high functional redundancy, and it has been suggested that *Aux/IAA* genes can have both similar and specific functions. The interactome map of Aux/IAA proteins in *Arabidopsis* shows that 29 *Aux/IAA* genes can interact with each other via 253 interactions and with 20 ARF proteins via 544 interactions (Luo et al., 2018). When *MsIAA32* is constitutively expressed in different tissues, the pleiotropic effects observed in *Arabidopsis* might be due to novel interactions with different sets of tissue-specific Aux/IAA or ARF proteins leading to changes in auxin perception. In *Arabidopsis*, *MsIAA32* could regulate non-glandular trichome formation, possibly through cyclin *AtCycB2-4*. Although the mutant phenotype of the *AtCycB2-4* gene in *Arabidopsis* is not available, other cyclin genes have been characterized which are involved in trichome formation (Schnittger et al., 2002). The effect of *MsIAA32* expression on other cyclin genes involved in trichome formation in *Arabidopsis* remains to be investigated. The ability of *MsIAA32* to control both non-glandular and glandular trichome formation indicates shared non-canonical Aux/IAA regulated auxin signaling mechanism among them. In contrast, *SlIAA15* studies indicate the involvement of canonical Aux/IAA mediated auxin signal pathways in the development of non-glandular and glandular trichomes. It is possible that both canonical and noncanonical Aux/IAA signaling pathways are connected and interact to fine tune auxin mediated development of trichomes in plants. Further research is required to understand such interactions and molecular mechanism of auxin triggered trichome development.

Our finding uncovers a new role for non-canonical *Aux/IAA* genes in glandular trichomes’ development in a non-model plant, spearmint, emphasizing the need to study these genes in different plant species for a deeper understanding of their functions in plant development. Secondary metabolites produced by glandular trichomes are important for plants’ response to abiotic and biotic stress and are economically important. Elucidating the genetic machinery underlying their formation will help to regulate their density and productivity.

## Data availability statement

The datasets presented in this study can be found in online repositories. **Supplementary Table S2** contains sequence data for all *MsIAAs* in **Figure 1A**. Sequences of *A. thaliana* and *Solanum lycopersicum* Aux/IAAs used in **Supplementary Figure S1** can be retrieved by the accession numbers shown in **Supplementary Table S3** from the NCBI database. *A. thaliana* and *Solanum lycopersicum* ARFs in **Supplementary Figure S7A** can be accessed in NCBI with accession numbers shown in **Supplementary Table S4**. *A. thaliana* R2R3-MYBs in **Supplementary Figure S8B** can be accessed in NCBI with accession numbers shown in **Supplementary Table S5**. The sequences of *MsIAA32, MsIAA3, MsIAA4, MsARF3* and *MsMYB36* are available in NCBI under sequence IDs MZ971168, MZ971169, MZ971170, MZ971171 and MZ971172 respectively.

## Author contributions

VR: Data curation, Formal analysis, Investigation, Methodology, Validation, Writing – original draft, Writing – review & editing. JS: Data curation, Investigation, Writing – review & editing. KN: Methodology, Writing – review & editing. RS: Conceptualization, Funding acquisition, Resources, Supervision, Writing – original draft, Writing – review & editing.
