# Supplementary material for: A non-canonical Aux/IAA gene MsIAA32 regulates peltate glandular trichome development in spearmint
Source: Front Plant Sci. 2024 Feb 5;15:1284125. doi: 10.3389/fpls.2024.1284125 (PMC10875047; doi:10.3389/fpls.2024.1284125)
Supplement: Supplementary file 1 [file DataSheet_1.pdf]

A non-canonical Aux/IAA gene *MsIAA32* regulates peltate glandular trichome development in spearmint

Supporting information

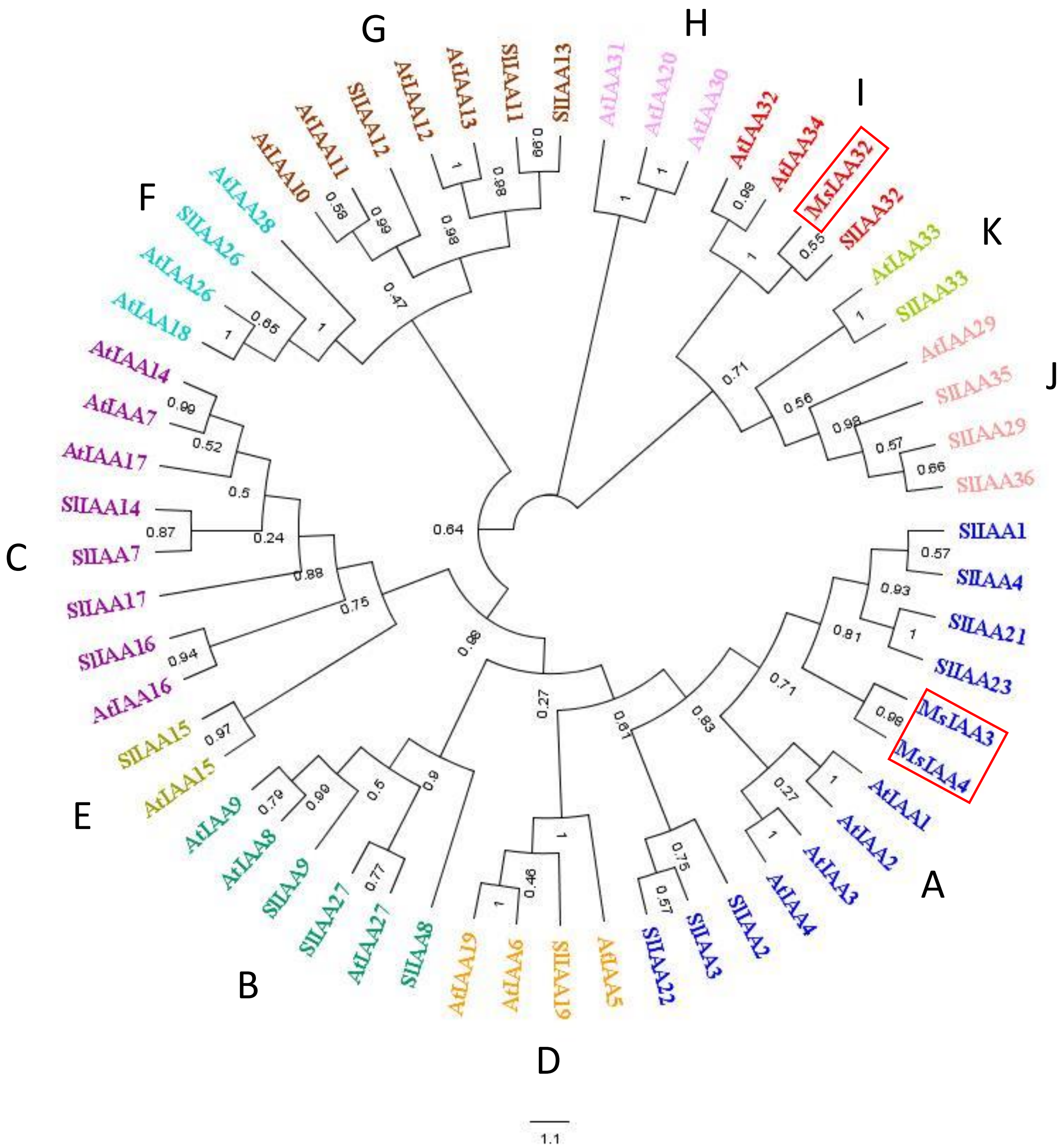

Fig. S1 Phylogenetic relationship between MsIAA32, MsIAA3 and MsIAA4 with other Aux/IAA proteins from Arabidopsis and tomato. The deduced full-length amino acid sequences were aligned by ClustalW and the phylogenetic tree was constructed by MEGA11 using Maximum Likelihood method. MsIAA32, MsIAA3 and MsIAA4 are highlighted with a red box. The numbers on the nodes represent bootstrap values from 1000 replicates. Accession number of sequences used in this tree can be found in Table S3.

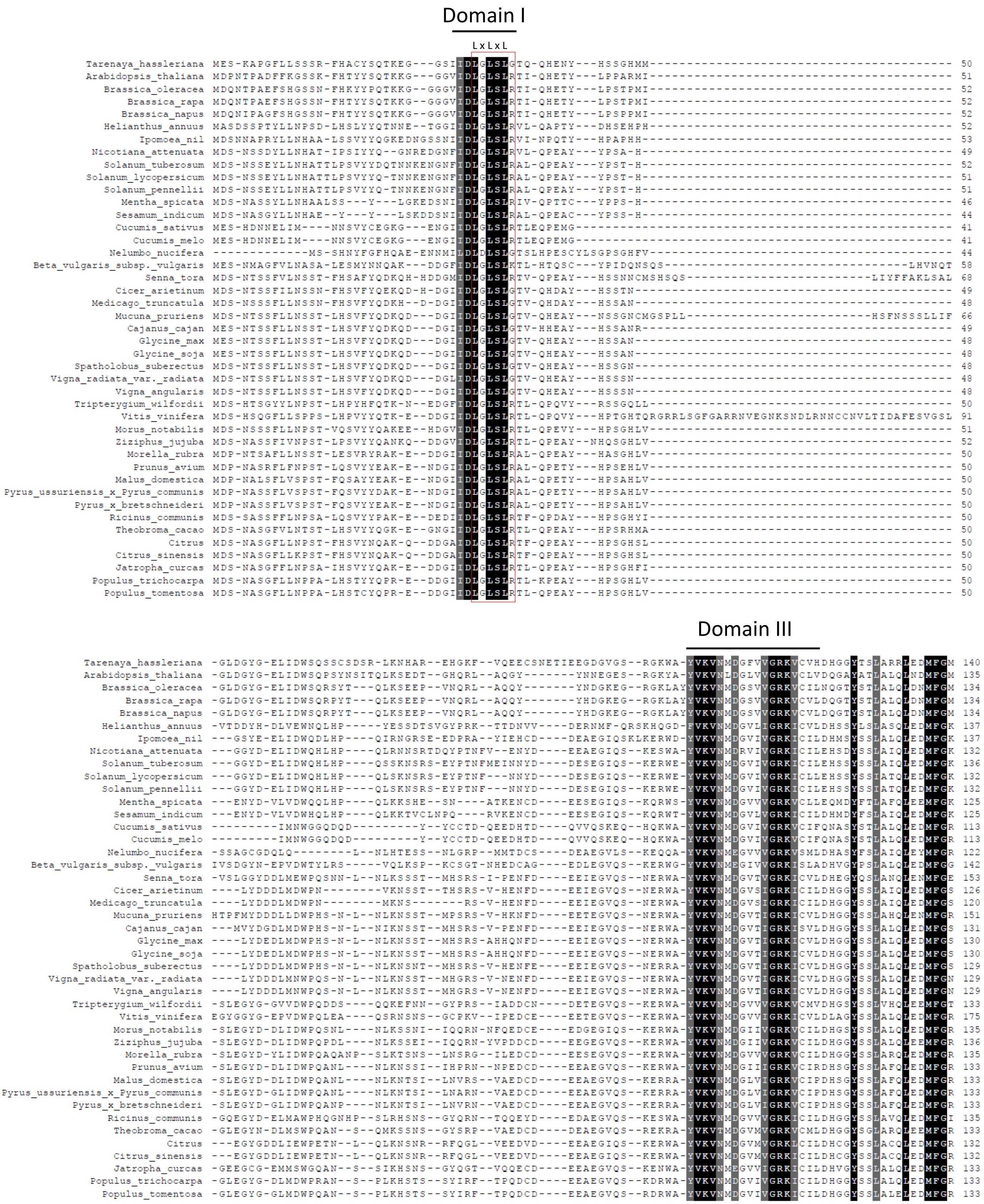
Domain I
L x L x L
Domain III

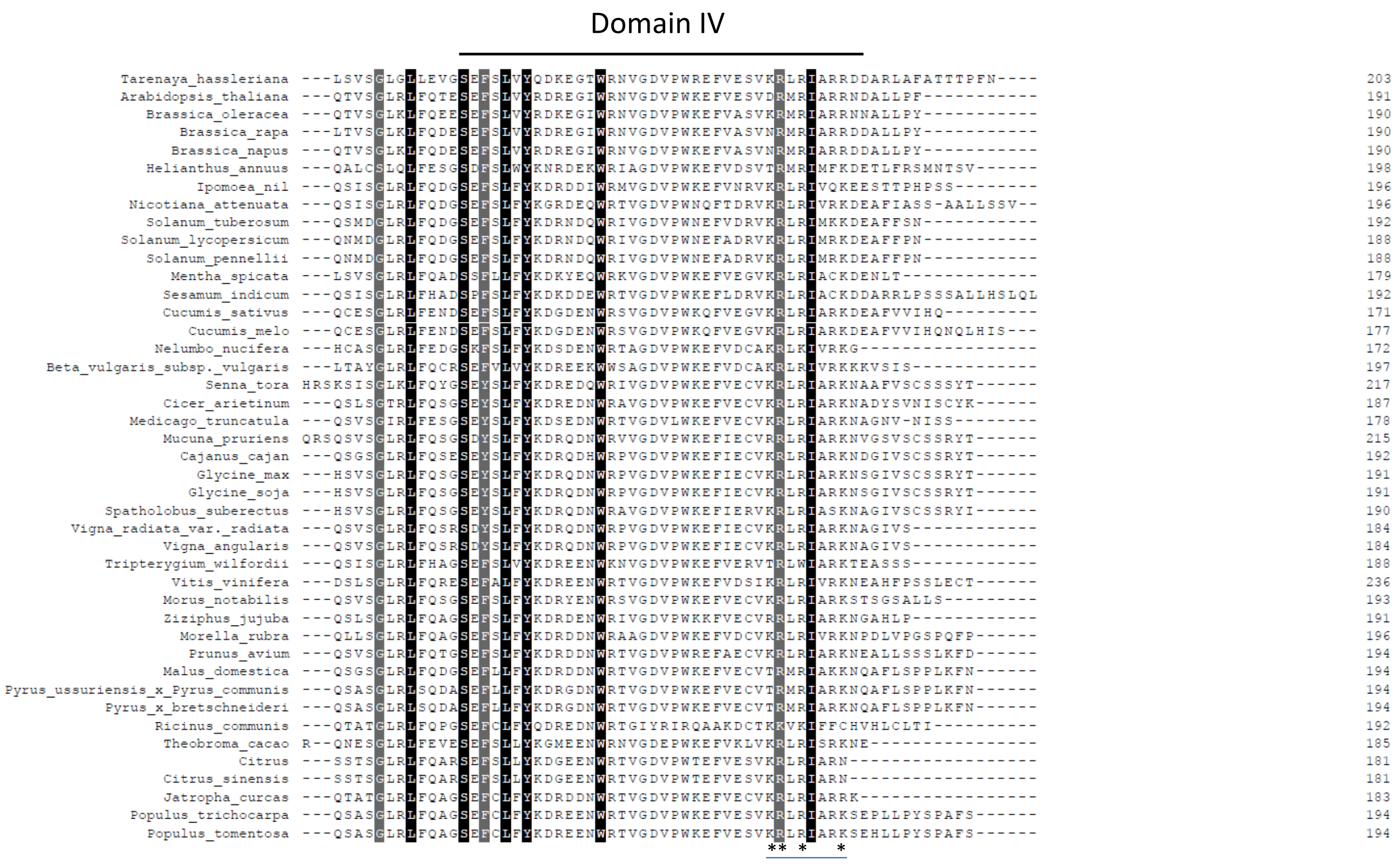

Fig. S2 Multiple sequence alignment of MsIAA32 with other IAA32 proteins from different plant species. Conserved amino acids are shaded in black. Domain I, Domain III and Domain IV are marked. Repression motif (LxLxL) is highlighted with a red box. NLS region is underlined in blue and the conserved amino acids (KR-R---K) are marked with *.

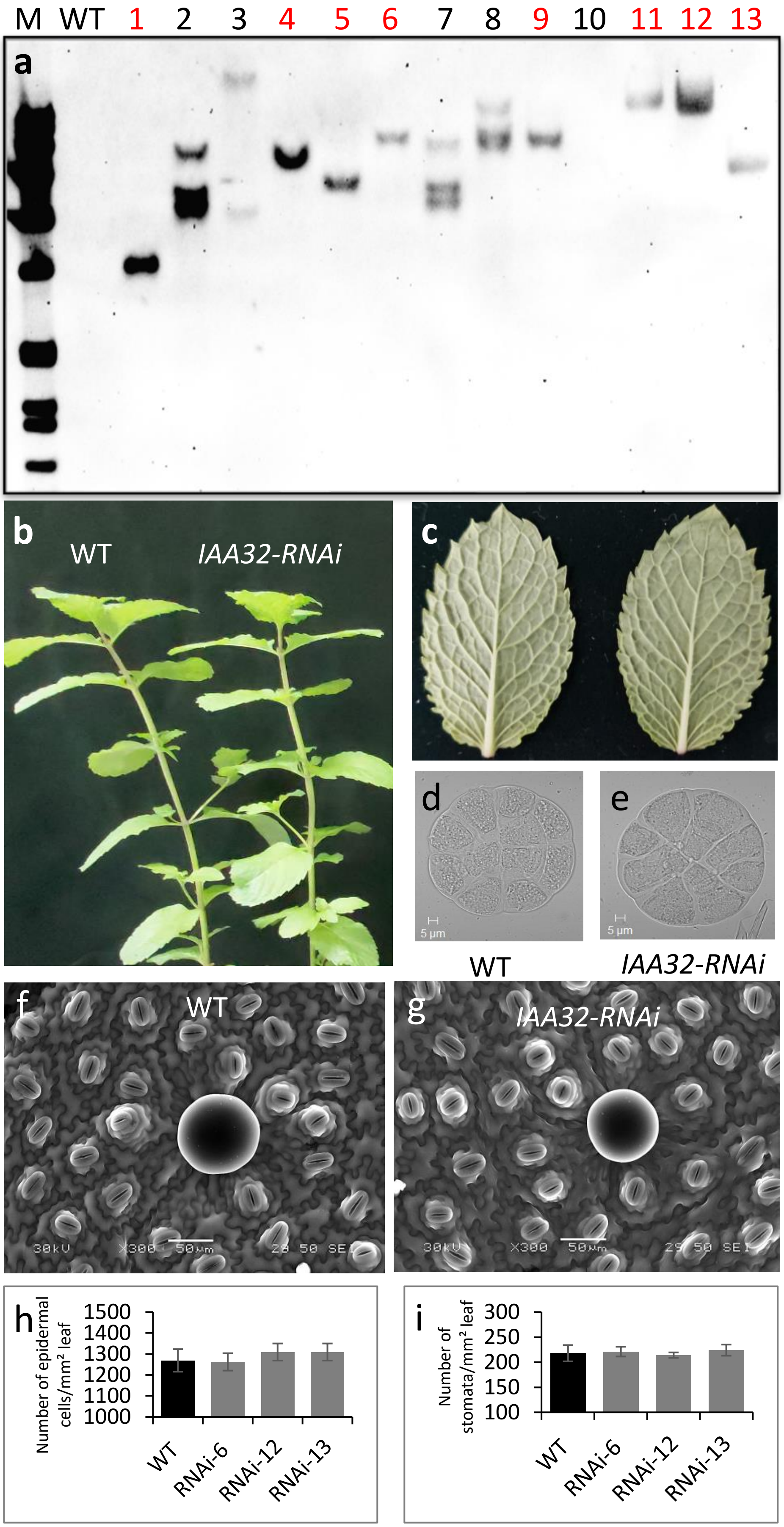

Fig. S3 Preliminary characterization of *MsIAA32*-RNAi lines. (a) Southern blot of *MsIAA32*-RNAi transgenics. M, marker; WT, wildtype; 1-13, *MsIAA32*-RNAi lines. WT plants and *MsIAA32*-RNAi plants showing similar plant growth and development **(b)**, leaf shape and size **(c)**, PGT shape and size **(d-g)**, Similar number of epidermal cells **(h)** and stomata **(i)** in WT and *MsIAA32*-RNAi plants .

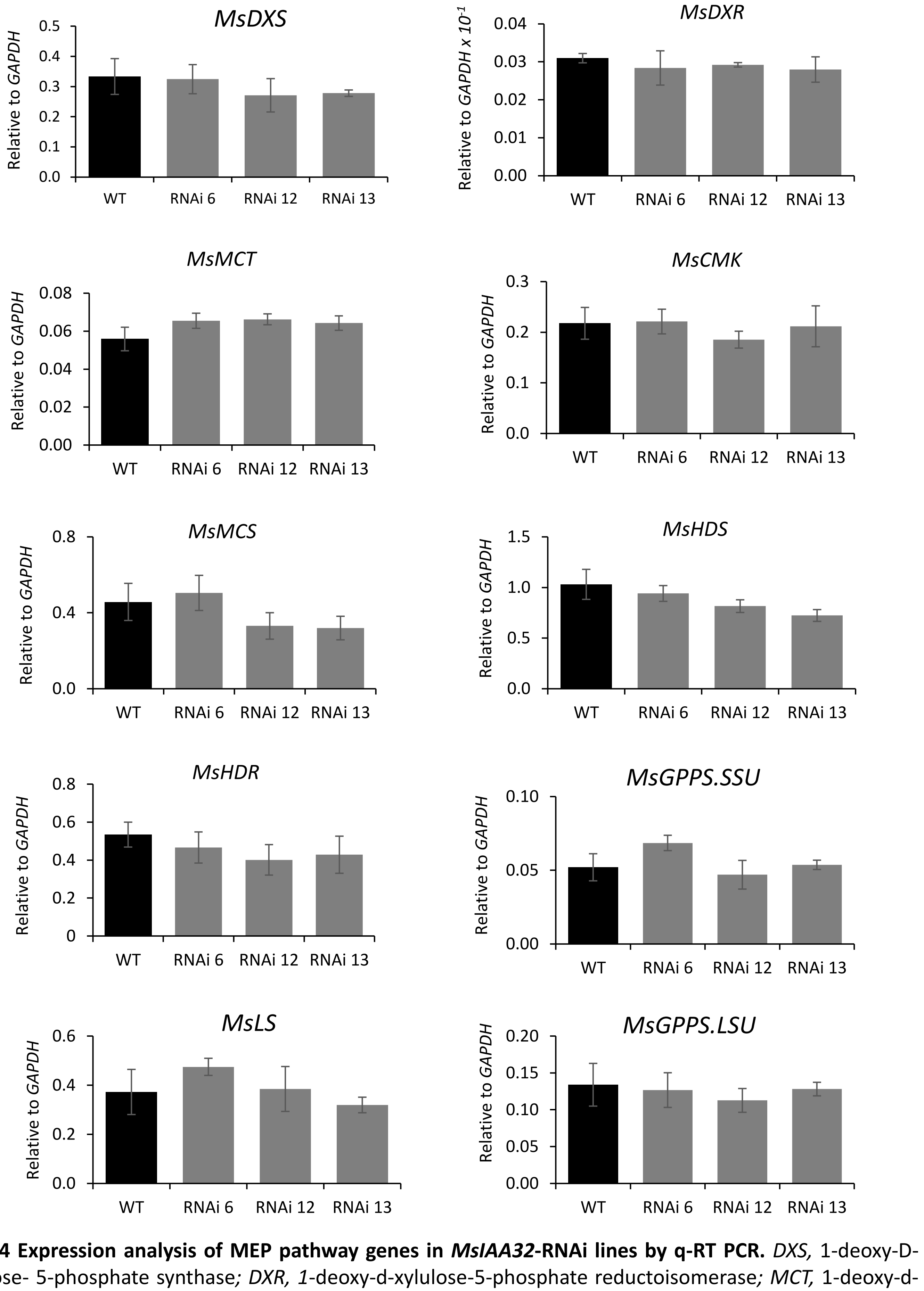

Fig. S4 Expression analysis of MEP pathway genes in *MsIAA32*-RNAi lines by q-RT PCR. *DXS,* 1-deoxy-D-xylulose- 5-phosphate synthase*; DXR, 1*-deoxy-d-xylulose-5-phosphate reductoisomerase*; MCT,* 1-deoxy-d-xylulose-5-phosphate reductoisomerase*; CMK,*4-(cytidine 5′-diphospho)-2-*C*-methyl-d-erythritol kinase*; MCS,* 2-*C*-methyl-d-erythritol 2,4-cyclodiphosphate synthase*; HDS,* 4-hydroxy-3-methylbut-2-enyl diphosphate synthase*; HDR,* 4-hydroxy-3-methylbut-2-enyl diphosphate reductase*; GPPS.SSU,* geranyl diphosphate synthase small subunit*; GPPS.LSU*, geranyl diphosphate synthase large subunit; *LS*, limonene synthase. Data are indicated as mean ± SD.

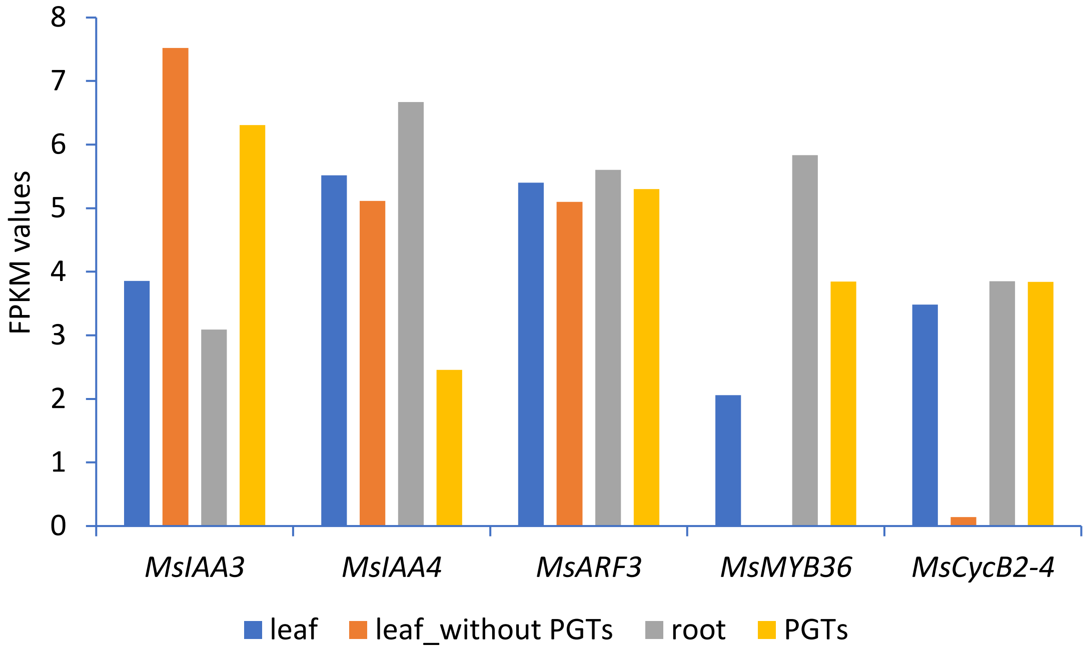

Fig. S5 FPKM values of genes. FPKM values of *MsIAA3, MsIAA4, MSARF3, MSMYB36* and *MsCycB2-4* along the tissues of leaf, leaf without PGTs, roots and PGTs. All the genes show expression in PGTs.

>MsIAA3

MECCDLNFKATELRLGLPGSDAVSPAKGSKRSSPELDDAGGVSSDRESAPPPPKAQIVGWPPVRSYRRNNIPANG AEAEVGIYVKVSMDGAPYLRKIDLKVYGGYSELLEALESMFKFTIGDYSEREGYKGSEYAPAYEDKDGDLMLVGDV PWEMFKSSCKRLRIMKGADARGLGCSV

>MsIAA4

MERIMANDLNLKATELRLGLPGSDQCDDVVSSAKNNKRASPETAEDSASNGVPAAKSAADRETAPAPKAQIVG WPPVKSYRKNNAAAATKAEAESGMFVKVSVDGAPYLRKIDIKLYKGYSELLDALEEMFKLSIGEYSEREGYKGSEY APAYEDKDGDLMLVGDVPWEMFMSSCKRLRIMRGADARGLGFAQ

Fig. S6 Sequence analysis of MsIAA3 and MsIAA4. (a) Amino acid sequences of MsIAA3 and MsIAA4. All the four conserved domains (I-IV) are highlighted in yellow with their respective conserved motifs in red.

a

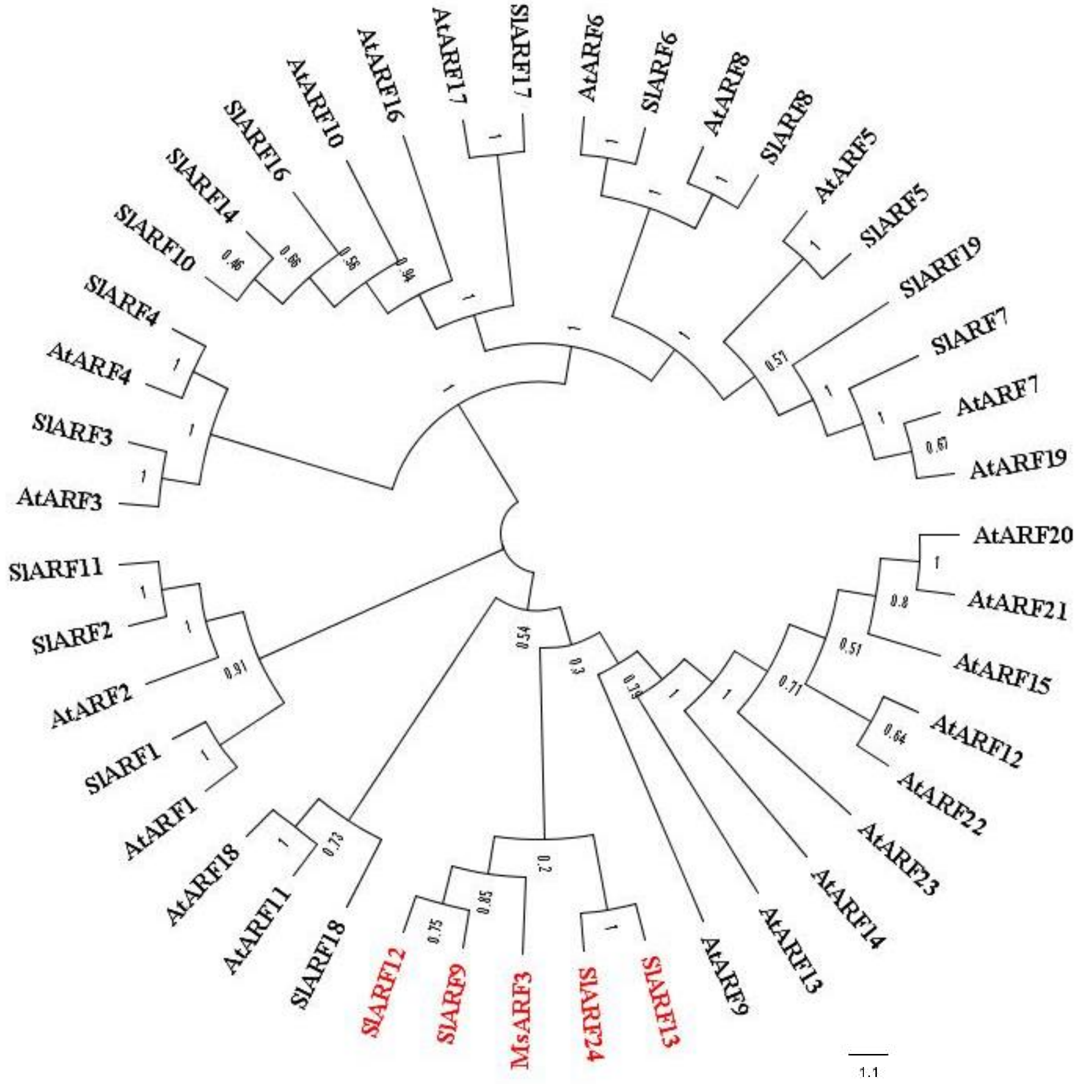

b

MANRVSLSQQQQYGGFPAEGGGGKDLMYEELWRACAGPLVEVPQVGEKVYYFPQGHMEQL

EASTNQELNQRIPLFNLPSKILCSVFNINLLAEVDTDEVYAQITLMPEADQTEPTSPDFS

YDEAPRPIVHSFCKVLTASDTSTHGGFSVLRKHANECLPPLDMNQQTPTQELIAKDLHGT B3

EWHFKHIFRGQPRRHLLTTGWSTFVTSKRLVAGDSFVFLRGENGKLRVGVRRHSRQQNSI

PSSVISSQSMHLGVLATASHALLTQTLFVVYYKPRTSQFIIGLNKYLEAMNQKFGVGMRF ARF

KMRFEGEDSPERRFSGTIVGVENISSHWEDSKWRSLKVQWDEPASIRRPERVSPWEIEPF

VPSIPTSLAPLPMLKHKRPRPHLEIPVSDNQMSQGVEGQKGNHMARYVAETDTIASPNMI

AEEGEVSKTASAWSVISNCSPPTSIKQNSTPISCMNERKPDTAATCRLFGFDLKNPSISP

LAENSSNISDDAAQEAEGCIKGQSKAPRMDAQSRNGHSSRSRTKVQMQGVAVGRAVDLTT Aux/IAA

LKGYDELIAELEEMFEIKGELQPRDKWEIVFTDNEGDMMLMGDDPWLEFVNMVRRIFICS

SQEVKKMKGSKLCEGTSLSLQHLGD

Fig. S7 Sequence and phylogenetic analysis of MsARF3. (a) Phylogenetic relationship between MsARF3 and other ARF proteins from Arabidopsis and tomato. The deduced full-length amino acid sequences were aligned by ClustalW and the phylogenetic tree was constructed by MEGA11 using Maximum Likelihood method. MsARF3 and the clade it falls within is highlighted in red, members of which are known to be repressors ARFs. The numbers on the nodes represent bootstrap values from 1000 replicates. Accession number of sequences used in this tree can be found in Table S4. **(b)** Amino acid sequence of MslARF3 showing the conserved domains.

a >MsMYB36
MGRAPCCDKANVKKGPWSPEEDAKLKEYIEKHGTGGNWIAFPQRAGLKRCGKSCRLRWLNYLRPNIKHGEFSDDEDRIICSLFARIGSRWSIIAGQLPGRTDNDIKNYWNTKLKKKLLGNIPQRRPLQQAISPPPLFPNLSSRIPSPYDYVSAASLRSFSTAAAAADPATAAALMSSGLPMHGNYNPETIYGGEASCSSSDSTNQIRENLGFQSYLSNGGVDDESQKFLINGTGRNGYDGKGGNGNCFGHENPMEYSMEEIKQLISTNHISTNLNFIVDEIKAEQGTLYY

b

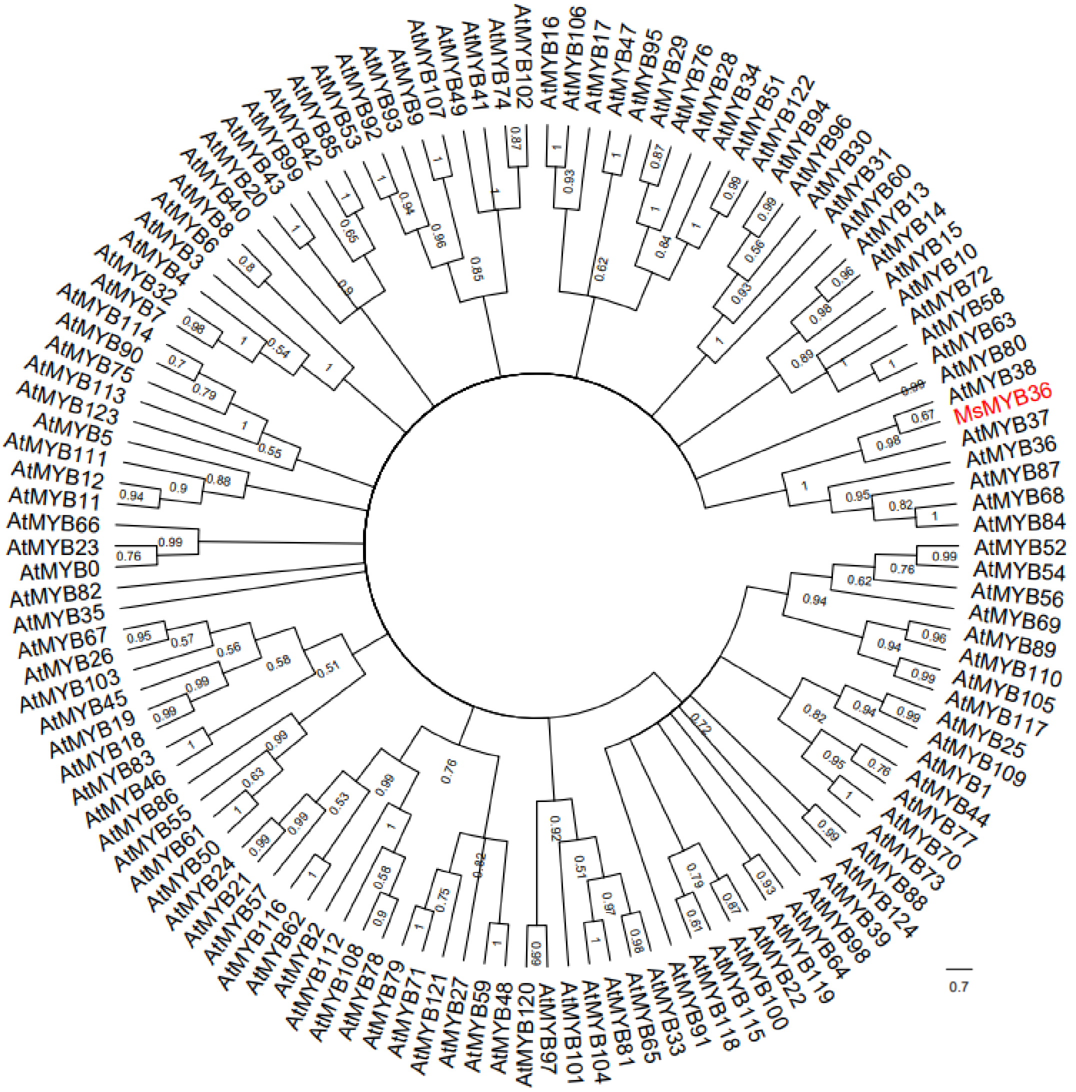

Fig. S8 Sequence and phylogenetic analysis of MsMYB36. (a) Protein sequence of MsMYB36. R2 and R3 repeats are highlighted in yellow and grey colours, respectively. Five conserved tryptophan residues are highlighted in red. **(b)** Phylogenetic relationship between MsMYB36 and other MYB proteins from Arabidopsis. The deduced full-length amino acid sequences were aligned by ClustalW and the phylogenetic tree was constructed by MEGA11 using Maximum Likelihood method. MsMYB36 is highlighted in red. The numbers on the nodes represent bootstrap values from 1000 replicates. Accession number of sequences used in this tree can be found in Supplemental Table 5.
